# Differential Associations of Early- and Late-Night Sleep with Functional Brain States Promoting Insight to Abstract Task Regularity

**DOI:** 10.1371/journal.pone.0009442

**Published:** 2010-02-26

**Authors:** Juliana Yordanova, Vasil Kolev, Ullrich Wagner, Rolf Verleger

**Affiliations:** 1 Department of Neurology, University of Lübeck, Lübeck, Germany; 2 Department of Neuroendocrinology, University of Lübeck, Lübeck, Germany; 3 Department of Cognitive Psychophysiology, Institute of Neurobiology, Bulgarian Academy of Sciences, Sofia, Bulgaria; 4 School of Psychology, Bangor University, Gwynedd, United Kingdom; Cuban Neuroscience Center, Cuba

## Abstract

**Background:**

Solving a task with insight has been associated with occipital and right-hemisphere activations. The present study tested the hypothesis if sleep-related alterations in functional activation states modulate the probability of insight into a hidden abstract regularity of a task.

**Methodology:**

State-dependent functional activation was measured by beta and alpha electroencephalographic (EEG) activity and spatial synchronization. Task-dependent functional activation was assessed by slow cortical potentials (SPs). EEG parameters during the performance of the Number Reduction Task (NRT) were compared between before sleep and after sleep sessions. In two different groups, the relevant sleep occurred either in the first or in the second half of the night, dominated by slow wave sleep (SWS) or by rapid eye movement (REM) sleep.

**Principal Findings:**

Changes in EEG parameters only occurred in the early-night group, not in the late-night group and indicated occipital and right-hemisphere functional alterations. These changes were associated with off-line consolidation of implicit task representations and with the amount of SWS but they did not predict subsequent insight. The gain of insight was, however, independently associated with changes of spectral beta and alpha measures only in those subjects from the two sleep groups who would subsequently comprehend the hidden regularity of the task. Insight-related enhancement of right frontal asymmetry after sleep did not depend on sleep stages.

**Significance:**

It is concluded that off-line restructuring of implicit information during sleep is accompanied by alterations of functional activation states after sleep. This mechanism is promoted by SWS but not by REM sleep and may contribute to attaining insight after sleep. Original neurophysiologic evidence is provided for alterations of the functional activation brain states after sleep. These alterations are associated with a decrease in controlled processing within the visual system and with an increase in the functional connectivity of the right hemisphere, and are supported by SWS in the first half of the night.

## Introduction

There are two general cognitive strategies that people use to solve problems. The first is the analytic strategy or ‘search’. It involves systematic conscious evaluation of intermediate problem states [1]. The second strategy involves insight [2]–[3]. Insight is the sudden awareness of the solution to a problem with little or no conscious access to the processing leading up to that solution [4]–[5].

Evidence suggests that the tendency to use analytic or insight strategies may be modulated by *basic modes of information processing* differing in the extent to which attention is focused on task-specific elements. Insight has been related to a tendency toward diffuse rather than focused (selective) attention. Less focused attention is suggested to decrease the strength of task-specific representations (close associations) and to increase task-irrelevant input (remote associations) whose activation may facilitate access, retrieval, and awareness of non-prepotent solutions thus promoting insight [6]–[7]. Insight strategy has also been associated with hemispheric asymmetry. Behavioural [6], [8], electrophysiological [9], and neuroimaging [9]–[11] studies suggest a special role for the right hemisphere activation in solving problems with insight.

### Sleep and Insight

**Figure 1:**
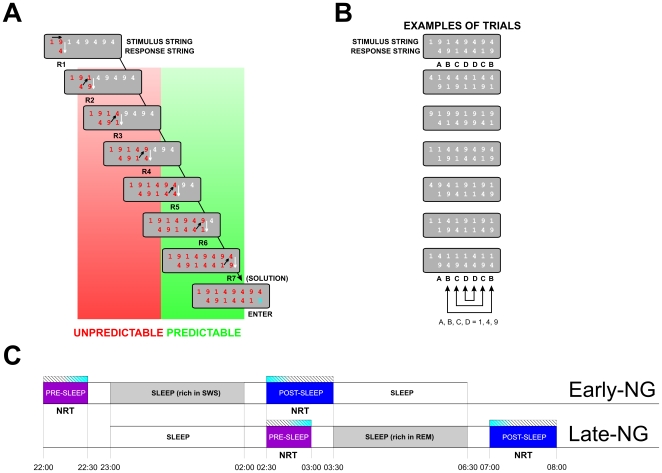
Experimental design of the study. (A) Schematic presentation of the number reduction task (NRT). Black arrows present the consecutive steps in NRT task performance (e.g., the first two numbers 1 and 9 in the stimulus string lead to response 4 (R1), then the same response (4) is compared with the next number from the stimulus string (1) leading to response 9 (R2), and so on). The final result is the last response (R7) marked with SOLUTION which is followed by Enter. According to their predictability during NRT processing, responses are divided into two response types: R2 to R4 – unpredictable and R5 to R7 – predictable. (B) Examples of trials demonstrating the abstract mirror structure of response strings (BCD - DCB) that characterizes each response string independently of the order of digits (1,4,9) comprising the string. (C) The experimental protocol. NRT pre-sleep and post-sleep sessions are marked for the two sleep groups (Early-NG and Late-NG). Hatching bars present the time period of EEG recording. Blue shadings during EEG recordings present the time windows used to extract 35 artifact-free epochs/sweeps for analysis.

By using the Number Reduction Task (NRT) [12]–[13] it has been demonstrated that sleep may lead to insight [14]. The NRT is a complex cognitive/procedural learning task where subjects transform in each trial a given digit string into a new digit string according to two simple transformation rules in order to determine a certain digit as the “solution digit” to the trial [12]–[15]. The NRT has two levels of organization, overt and covert. As detailed in Methods and illustrated in Fig. 1A,B, each trial of the NRT consists of a string of several digits (stimulus string). At the overt level, subjects have to process the digits and produce consecutive responses following two operational rules, which creates the response string (Fig. 1A). The covert level of NRT organization is that unmentioned to the subjects, all strings are generated according to an abstract regularity according to which the last three responses in a response string always mirror symmetrically the preceding three responses, so that the second response in each trial is identical to the final solution (Fig. 1A,B, the abstract code is presented by ABCDDCB). This regularity is abstract because the actual digit strings and responses change from trial to trial (Fig. 1B). This regularity can be discovered and applied consciously thereafter reducing the number of required responses from seven to two, which indicates generation of explicit knowledge about the NRT structure and is a marker of insight [15]. Notably, in the NRT, implicit knowledge about the hidden regularity also can be acquired. This implicit learning has been verified by the speeding of responses that can be predicted relative to those that cannot be predicted by that regularity (as indicated in Fig. 1A) although the subjects remain unaware of the presence of any structure [13], [15]–[18].

Sleep studies have found that more than twice as many subjects gained insight into the hidden regularity at retest in Wagner et al. 's study (2004) [14] if they had slept after the initial training than if they had not. To account for this result, Wagner et al. (2004) considered off-line memory consolidation and re-organization as the principal mechanism promoted by sleep. This is in accordance with evidence that sleep supports the consolidation of both explicit and implicit memories (revs. [19]–[25]).

However, about 20% of the individuals can solve the NRT directly with insight even when no periods allowing memory consolidation are inserted between learning and retest sessions [14], [16]–[17], [26]. Also, in the NRT study of Yordanova et al. (2008) [16], the level of pre-sleep implicit knowledge was not a factor that *absolutely* determined whether insight would be developed across subsequent sleep, since half of the subjects who gained explicit insight into the hidden NRT rule after sleep had *not* acquired implicit knowledge before sleep. Thus, memory re-organization may be an important but not an absolute predictor of insight.

Instead, insight solutions in tasks with solving verbal anagrams have been shown to be preceded by specific functional activation states of the brain [27]–[28]. The tendency to use insight strategies can be therefore modulated by *individual activation traits* [29] as well as by *ongoing functional activations* of the brain promoting attentional diffusion and right-lateralized hemispheric asymmetry [27]–[28].

The aim of the present study was (1) to explore if sleep modulates functional activation states that may support differentially (either positively or negatively) insight into the rule of the NRT, and (2) to analyze the role of sleep stages, slow wave sleep (SWS) and rapid eye movement (REM) sleep, in these processes. To assess functional activation states, brain electric (EEG) signals were recorded and analyzed while subjects from two sleep groups (early-night and late-night) performed the NRT before and after a retention period of 3 hours full of SWS in the early half of the night or of REM sleep in the late half of the night [30]–[34] – Fig. 1C.

### EEG Markers of Insight: Hypotheses of the Present Study

In the present study, activation patterns are defined as state-dependent and task-dependent. A *state-dependent* activation creates a neural environment maintaining similar processing conditions for different task elements for a certain period of time. A *task-dependent* activation is controlled by executive brain systems and selectively enhances or inhibits region-specific networks to optimize transiently the processing of task-specific elements. Here, it was tested if markers of state-dependent activations known to promote the insight mode of processing associated with diffuse visual attention at occipital regions and with right-hemisphere activation [9]–[10], [27]–[28], would appear after sleep such as to induce insight also in the NRT. Another question was if such activations would emerge as non-specific functional states, or they might be determined by task-specific information. To evaluate the effects of task-specific information, comparisons were made between the processing of different response types in the NRT, i.e., between responses that can or cannot be predicted by the hidden regularity. The assumption was that state-dependent functional activations would be information-nonspecific and should not differentiate task-specific material. The following electrophysiological parameters were used to address these issues:

#### Alpha and beta EEG oscillations

Prior research suggests that functional brain states underlying different strategies of problem solving can be assessed by alpha (alpha-1, 7–10 Hz and alpha-2, 10–13 Hz) and beta (14–18 Hz) EEG oscillations. Enhanced occipital beta has been associated with an excitatory mechanism of selective attention [35]–[36]. In contrast, enhanced occipital alpha has been related to an inhibitory gating mechanism regulating the intake of information in task-irrelevant visual areas [37]–[42]. Accordingly, increased posterior alpha is accompanied by less efficient visual perception, in contrast to decreased posterior alpha leading to improved perception [43]–[47]. Most recent data indicate that the inhibitory mechanisms associated with enhanced posterior alpha support primarily the continuous maintenance of optimal activation level for target processing, whereas facilitatory processes reflected by alpha decrease subserve transient activations for target anticipation [41]. Although the exact functional significance of alpha dynamics is currently being elucidated (e.g., refs. [38]–[39], [48]), a consistent finding relevant to the present study is that subjects tending to solve problems analytically had increased occipital beta (i.e., greater focused visual attention associated with more neural activity to task-specific information) and increased occipital alpha-2 (more inhibition of brain areas processing non-attended visual information) than subjects tending to solve problems with insights [28]. In addition, the insight strategy was accompanied by hemispheric asymmetry in alpha-1 activity indicative for a greater right- than left-hemisphere excitability [27]–[28], [49]. If sleep supports the insight mode by modulating functional activation states, less occipital beta and less occipital alpha-2 EEG activity, as well as less alpha-1 activity over the right hemisphere can be expected during NRT performance after sleep than before sleep. Also, if induced by functional brain states and being information-nonspecific, these effects would not differ between unpredictable and predictable items.

#### Spatial synchronization

Increased functional activation induces facilitated communication among functionally relevant cortical regions [50]–[55]. Specifically, spatial synchronization in the theta frequency range (4–7 Hz) has been found to reflect long-range connectivity between distant cortical regions [52]–[53]. If sleep supports insight-related brain states, a stronger synchronization would emerge within occipital and within right-hemisphere regions after than before sleep, with this patterns being again similar for unpredictable and predictable items in the NRT.

#### Slow EEG shifts

Slow potentials (SPs) appear as positive or negative DC shifts of the ongoing EEG during task processing lasting up to several seconds [17], [56]–[57]. It has been demonstrated that negative SPs index functional activation of cortical regions involved in controlled task processing [57]–[58]. Most recent fMRI research conducted in combination with SP analysis has confirmed that the topography of the slow waves reveals the pattern of controlled activation (higher excitability) and deactivation (reduced excitability) of underlying cortical modules [59]–[60]. SPs were measured here to characterize task-dependent transient activations after sleep and their associations with functional brain states at occipital and right-hemisphere regions. A detailed topography analysis of SPs from the same task and sample is presented elsewhere [18]. To separate the effects of SWS and REM sleep, the procedure of night-half comparison was applied [16].

A summary of the analytic design, parameters and insight-promoting effects being tested in the present study are presented schematically in Table 1.

**Table 1:** Functional modes promoting insight: summary of analytic design and parameters.

		DIFFUSE VISUAL ATTENTION		RIGHT HEMISPHERE ACTIVATION	
Functional mechanisms	EEG Correlates	Parameter	Insight promoting effect	Parameter	Insight promoting effect
**State-dependentactivation**	EEG oscillations	Occipito-parietal beta power Occipito-parietal alpha-2 power	Decrease Decrease	Fronto-parietal alpha and beta power in the RH and in the LH	Decrease in the RH
**Spatialconnectivity**	Spatial synchronization	Occipito-parietal phase-synchronization (PLV)	Increase	Phase-synchronization in the RH and in the LH	Increase in the RH
**Task-dependentactivation**	Slow cortical potentials	Occipito-parietal SP amplitudes	Positivization of negative SPs	SP amplitudes in the RH and in the LH	More negativity in the RH

SP, slow cortical potential, PLV, phase-locking value (spatial synchronization), RH, right hemisphere, LH, left hemisphere.

## Materials and Methods

### Ethics Statement

This research was approved by the ethics committee of the University of Lübeck, Lübeck, Germany. Informed written consent was obtained from all subjects prior to the study.

### Subjects

The sample reported in the study of Yordanova et al. (2008) [16] (see also ref. [18]) was used for the present analysis. Fifty-five healthy students (18–28 year old) without any history of sleep disturbances or psychiatric or neurological disorders participated in the experiments. All subjects spent an adaptation night in the sleep laboratory including placement of electrodes. Subjects were paid for their participation and gave informed written consent prior to the study, which was approved by the local ethics committee. From this data set (29 subjects from the early-night group and 26 subjects from the late-night group reported in ref. [16]), 4 subjects from the early-night group and 3 subjects from the late-night group were excluded because of lacking or low-quality EEG records during NRT performance before or after sleep. Thus, for the present analysis a total of 48 subjects were used for statistical comparisons (25 from the early-night group and 23 from the late-night group).

### Task

The task is illustrated in Fig. 1A. It was the same version of the NRT as described previously in ref. [14]. On each trial, a different string of eight digits was presented. Each string was composed of the digits 1, 4, and 9. For each string, subjects had to determine a digit defined as the final result of the task trial (Solution). This could be achieved by sequentially processing pairs of digits from left to right according to two simple rules: (1) The “identity rule” states that the result of two identical digits is the same digit (e.g., 4 and 4 gives 4, see Fig. 1A – Response 5, R5). (2) The “difference rule” states that the result of two non-identical digits is the remaining third digit (e.g., 1 and 9 gives 4, see Fig. 1A – R1-R4, R6-R7).

The 1, 2, and 3 keys on the PC numeric pad were labeled accordingly 1, 4, and 9 and served as response keys. The entered responses appeared on the screen and remained there until the end of the trial, thereby forming a response sequence below the stimulus sequence. To produce the first response, comparisons are made between the first and the second digits from the stimulus string (Fig. 1A – R1). After processing the first two digits, comparisons are made between this result (appearing in the response string) and the next digit from the stimulus string, then between the result of this new processing and the next digit, and so on (Fig. 1A). Thus, applying the two rules, subjects generated a string of seven responses, with the last one (R7) indicating the final result (Solution) to be confirmed by pressing the “Enter” key on the numeric pad. The time for any single response was limited to 4 s and to a total of 12 s for all responses until pressing ”Enter”. Pressing the ”Enter” key was followed by a change of color of the entered final response on the screen, from red to blue (Fig. 1A – R7-Enter). After another 1-s period, feedback was provided. In case of a correct final result, all digits on the screen, in addition to the final one, changed their color to blue, whereas the red German word “Wrong” appeared on the screen in case of an incorrect solution. The screen was cleared after another 0.5 s, and the next trial started.

Instructions stated that only the final result was to be determined for each trial and this could be done at any time. Importantly, unmentioned to the subjects, all strings were generated according to the same underlying regularity, which, if discerned, allowed an early determination of the solution. Specifically, as shown on Fig. 1B, all response sequences had the form ABCDDCB (with A, B, C, and D representing one of the digits 1, 4, or 9), i.e. the last three responses always mirrored the preceding three responses, so that the second response in each trial coincided with the final solution. Thus, when gaining insight into this regularity, participants abruptly cut short sequential responding by pressing the ”Enter” key already after the second response, whereupon the trial was finished and the next trial started. Note that this regularity is abstract because the actual digit strings and responses changed from trial to trial. Thus, discovery of the rule cannot simply be based on repetition of the same digits or the same finger movements in all trials.

Reaction times (RTs) were measured continuously during task performance, separately for each response in the response string. RT of the first response (R1) was measured as the time from string appearance to the first key press. The RTs of the other responses (R2, R3, R4, R5, R6, R7, Enter) were measured as the time between the previous and the current key press.

### Experimental Procedure

The experimental design is presented in Fig. 1C. Subjects were tested individually in a sound-attenuated room. As in the previous study of Wagner et al. (2004) [14], subjects performed a pre-sleep session of initial practice comprising of 3 task blocks and a post-sleep retest session of 10 task blocks, with 30 trials in each block. Insight was automatically identified by the program when at least 24 correct short-cuts within the same block occurred, in which case the task was terminated. Initial practice was preceded by extensive standardized instructions given on the computer screen, which included a short block of 10 task trials. To assure correct understanding of the “identity” and “difference” rule, this block was repeated as long as the subject performed the 10 trials without mistake. To investigate the effects of different sleep phases, the interval between initial training and retest was filled with three hours of sleep either in the early night, containing high amounts of SWS, or in the late night, containing high amounts of REM sleep (Fig. 1C).

In the early-night group (Early-NG), subjects reported to the laboratory at about 21:00 h. After placement of electrodes, they performed the three blocks of initial training (including preceding computer-guided instructions) at about 22:00 h and thereafter went to bed at about 23:00 h. After three hours of sleep in the early night they were awakened to perform the 10 blocks of NRT retesting. Subjects in the late-night group (Late-NG) reported to the laboratory at about 22:00 h and, after placement of electrodes, first slept for three hours in the early night before performing the initial training at about 2:30 h. Then, they slept again for another three hours in the late night (about 4:00 h – 7:00 h), followed by retesting in the morning. In all conditions, sessions also included performance in a short simple choice-response task unrelated to the present study, taking place immediately before and after sleep (i.e. after initial NRT training and before NRT retesting).

Subjects were only awakened from light sleep stages 1 or 2 to avoid cognitive disturbances that can occur after awakenings from SWS or REM sleep. As an additional control, subjective levels of sleepiness, activation, boredom, concentration, and motivation were assessed on 5-point scales immediately before and after each session of initial training and retest [14].

After NRT retesting, subjects filled in a questionnaire related to their explicit knowledge of the task structure (beginning with open questions, followed by closed questions) as well as possible strategies used during task performance. An additional behavioral test comprised a speeded task in which 15 different strings were presented and subjects had to indicate the final result to each string within 2 s after string presentation.

### Sleep EEG Recording and Analyses

Sleep was recorded polysomnographically, including EEG recordings from the left and right central sites (C3, C4), horizontal and vertical EOG, and EMG from chin electrodes. Sleep stages S1, S2, S3, S4, and REM sleep were classified in 30-second epochs according to ref. [61]. SWS was calculated as the sum of time spent in sleep stages S3 and S4.

### Task-Related EEG Recording and Analyses

During the NRT performance, EEG was recorded continuously from 28 scalp electrodes located on positions AF3, AF4, F7, F3, Fz, F4, F8, FC5, FC1, FC2, FC6, T7, C3, Cz, C4, T8, CP5, CP1, CP2, CP6, P7, P3, Pz, P4, P8, PO3, PO4, and Oz according to the 10–20 International system. The vertical electrooculogram (VEOG) was recorded from electrodes placed above and below the left eye. The horizontal electrooculogram (HEOG) was recorded from electrodes attached to the outer canthi of the eyes. All electrode sites were referenced to linked mastoids. EEG and EOG signals were amplified by using a Neuroscan Synamps, with impedances maintained below 10 kOhms. EEG and EOG were filtered within the pass-band 0.03–70 Hz and sampled with a frequency of 250 Hz. Times of EEG recording are illustrated on Fig. 1C by hatching bars.

Data processing was performed with Brain Vision Analyzer software (Brain Products GmbH, Gilching, version 1.05). EEG traces were visually inspected for gross EOG and EMG artifacts. Contaminated trials were discarded along with records exceeding ±50 µV. Slight horizontal and vertical eye movements preserved in the accepted trials were corrected by means of a linear regression method for EOG correction [62]. In all analyses described in the following, for evaluation of EEG changes during task execution, 30 EEG segments were collected for two conditions (end of pre-sleep practice and immediately after sleep, see Fig. 1C, fading-blue bars).

#### EEG analysis

Segments with a length of 800 ms were collected before each response (R1, R2, …, R7). Segments were tapered by Hanning windows with a length of 20% from the epoch boundaries. By using a fast Fourier transform (FFT), the power spectrum was obtained, with a frequency resolution of 0.977 Hz. Single spectra were averaged and mean values were calculated for each single frequency from 2 to 18 Hz and for the frequency bands alpha-1 = 7−10 Hz, alpha-2 = 10−13 Hz, and beta = 14−18 Hz. Statistical analyses were performed for each frequency band. In order to normalize distributions, a log10-transform was applied to the data (e.g., ref. [63]).

#### Spatial phase-locking

EEG segments of 1600 ms centered at the moment of response production (R1, R2, …, R7) were used for analysis. Before the estimation of phase-synchronization, to achieve a reference-free evaluation, the current source density (CSD) at each electrode position was obtained by applying the spherical Laplace operator to the voltage distribution on the surface of the scalp [64]. This procedure was characterized by the following parameters: order of splines m = 4, and the maximum degree of the Legendre polynomials n = 10, with a precision of 2.72^−5^ [65].

Time-frequency transforms were obtained by the application of complex-valued Morlet wavelets [66], which are Gaussian in both the time and frequency domains. Complex Morlet wavelets *w* can be generated in the time domain for different frequencies, *f*, according to the equation:



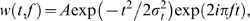



where *t* is time, 
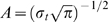
, 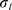 is the wavelet duration, and 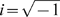.

For analysis, a ratio of f_0_/σ*_f_* = 5.5 was chosen, where f_0_ is the central frequency and σ*_f_* is the width of the Gaussian shape in the frequency domain. The choice of the ratio f_0_/σ*_f_* was oriented to the expected slower phase-locked components present in the response-related potentials, which had an effect on the shape of the Morlet wavelet and decreased its decay (e.g., ref. [55]). The analysis was performed for each single sweep, with central frequencies varying by 0.5 Hz from 4 Hz to 30 Hz. For different f_0,_ time and frequency resolutions can be calculated as 2σ*_t_* and 2σ*_f_*, respectively [67]. σ*_t_* and σ*_f_* are related by the equation σ*_t_* = 1/(2πσ*_f_*). The signal in each single sweep was convolved with the complex Morlet wavelet designated for f_0_. The Morlet wavelet was normalized by subtracting the mean value of the baseline period (800 to 600 ms before key press). The complex phase value was then computed at frequency f_0_, for each electrode, each time bin and each single sweep by dividing the result of the convolution by the magnitude of this result [67]–[68].

Subsequently, a phase-locking value (PLV) was computed for each time-point *t* and trial *j* as:



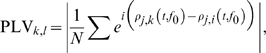



where *N* is the number of single sweeps, *k* and *l* are the index for the pair of electrodes to be compared, and 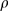
is the instantaneous phase of the signal. PLV*_k,l_* results in a real value between one (constant phase difference) and zero (random phase difference). These values were normalized by subtracting the mean value of the baseline period (800 to 600 ms before key press) and dividing by the standard deviation of this time window [68]. This procedure is similar to the z-score transformation and is known as a standardizing procedure that allows a reliable comparison between highly variable data. For this analysis, sweeps obtained for different response types were pooled together.

Statistical evaluations were performed only for PLV that significantly differed from random noise. To extract these PLV measures, a statistical randomization technique was used. The central epoch between 400 ms before and 400 ms after key press was divided into eight equal time windows, 100 ms each. For the respective time window and electrode pair, the PLV mean value was calculated and compared with the maximal or minimal value (for evaluation of synchronization and desynchronization accordingly) obtained in the whole epoch resulting from the same set of single sweeps which were randomly shuffled 200 times [69]. A statistically significant value was accepted if the measured parameters were larger (for synchronization) or smaller (for desynchronization) than the respective maximal or minimal values in the whole epoch after shuffling. This criterion is much more conservative than used in other studies, in which significance is accepted if the PLV is above 97.5 percentile of the distribution of shuffled maximal values (for synchronization) or below 2.5 percentile of the distribution of shuffled minimal values (for desynchronization, see, e.g. ref. [70]).

Although PLV was computed for all electrode pairs, it was analyzed statistically for electrode pairs selected to reflect inter-hemispheric phase relations in occipital regions (PO3-PO4), and intra-hemispheric phase relations in occipital, parietal, central and frontal regions within the left and the right hemisphere (PO3-CP5, PO3-C3, PO3-FC5, PO4-CP6, PO4-C4, PO4-FC6). To reflect the activation of executive control regions, the phase-locking between mid-frontal (Fz) and central motor regions (contra-lateral to the responding hand, C3, and ipsi-lateral, C4) was also analyzed [50], [71]. These were Fz-C3, Fz-C4, C3-C4, and FC5-FC6.

#### Analysis of slow potentials

**Figure 2:**
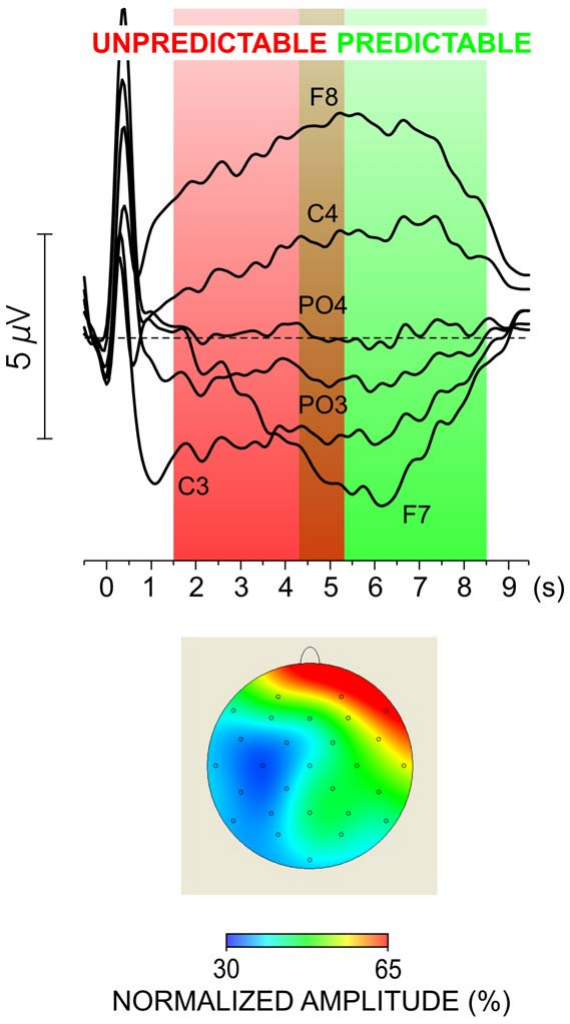
Temporal and spatial characteristics of slow potentials (SPs) in the NRT. Time course of SPs at selected electrodes (top) demonstrates typical SP topography shown on the map (below). Positivity upwards. String appears at time 0. The time epochs of unpredictable response processing R2-R4 is marked in red, the time epochs of predictable response processing R5-R7 is marked in green. The overlapping of epochs of unpredictable and predictable response processing is due to reaction time variability. Normalized amplitude presented on the map is obtained by means of min-max procedure across electrodes.

SPs were obtained after extracting 9.5-s EEG epochs triggered by the moment of string appearance, with a 0.5-s pre-stimulus baseline. As shown in Fig. 2, for each subject, condition, and electrode, mean values were measured for 8 consecutive 1-s time windows starting 1.5 s after stimulus. The choice of this starting point was to avoid stimulus related phenomena such as P300 or other slow ERP components. For analysis, difference waves were produced by subtracting pre-sleep from post-sleep SPs.

### Statistical Analyses

In the present experimental design, seven consecutive responses (R1 to R7) were produced (Fig. 1A) designated as a factor Response Number (R1 vs. R2 vs. R3 vs. R4 vs. R5 vs. R6. vs. R7). According to the mirror rule, R2, R3, and R4 represent the unpredictable responses, and R5, R6 and R7 represent the predictable responses, thus forming the factor Response Type (unpredictable vs. predictable). Parameters were subjected to analysis of variance (ANOVA), as will be detailed.

#### Occipito-parietal spectral EEG

To test the hypothesis of insight-related markers of *visual attention*, spectral EEG power values of alpha-2 (10–13 Hz) and beta (14–18 Hz) activities were analyzed at the occipital PO3, Oz, and PO4 electrodes for the epoch of 800 ms preceding each response (R1 to R7). The ANOVA design included two between-subjects variables, Sleep Group (Early-NG vs. Late-NG) and Processing Strategy (Solvers vs. Non-solvers). Solvers are the individuals who gained insight to NRT mirror rule after sleep, in contrast to non-solvers. The within-subjects variables were Session with two levels (pre-sleep vs. post-sleep), Response Number with seven levels (R1, R2, …, R7), and Electrode with three levels (PO3, Oz, and PO4). Out of hypothesis power values of alpha-1 were subjected to the same analysis.

#### Asymmetric effects on spectral EEG

To test the hypothesis of insight-related markers of right vs. left asymmetry, alpha-1, alpha-2 and beta measures of spectral EEG at frontal and parietal sites were subjected to a Sleep Group x Processing Strategy x Session x Response Number x Laterality (right vs. left) ANOVA. Central and centro-parietal electrodes were not included in this analysis because of the specific design of the NRT, which required movements with the right hand and could induce a motor-related decrease of alpha and beta activity over the sensorimotor cortical regions of the left hemisphere [72]. Thus, asymmetry was evaluated by using right and left frontal (F4 and F3) and right and left parietal (P4 and P3) electrodes. To avoid confounds from the specific scalp distribution (mainly of alpha activity predominantly at posterior sites), asymmetry analyses were done separately for the frontal and parietal locations.

#### Spatial synchronization

PLV was analyzed for inter-hemispheric and intra-hemispheric electrodes within the left and the right hemisphere (see 2.6) for the theta frequency scale (f_0_ = 4.92 Hz, approx. band limits 4–7.38 Hz). Because data distribution deviated from the normal one, statistical analyses were performed by using nonparametric Wilcoxon-Wilcox test for each selected electrode pair to evaluate the effects of early and late sleep and processing strategy.

#### Slow cortical potentials

Figure 2 shows that SPs had a characteristic spatial pattern in the NRT, with negative SPs distributed primarily over the left hemisphere, and positive SPs distributed over the right hemisphere [18]. Accordingly, relevant regions of interest (ROI) with electrodes from occipital-parietal regions, left (LOP) and right (ROP), were used for the evaluation of occipital SPs. For analysis of hemisphere asymmetry effects, left and right fronto-temporal (LFT, RFT) and central (LC, RC) groups of electrodes were used (details in ref. [18]). The mid-frontal-central ROI (MFC) was analyzed separately to reflect the activation regions engaged in controlled executive monitoring. Statistical evaluation was performed on difference values obtained by subtracting SPs before sleep from SPs after sleep. The ANOVA design included two between-subjects variables, Sleep Group (Early-NG vs. Late-NG) and Processing Strategy (Solvers vs. Non-solvers), and within-subjects variables, Response Type (unpredictable vs. predictable) and ROI (LOP vs. ROP, for assessing occipital effects) or Laterality (left vs. right) x ROI (fronto-temporal vs. central, for assessing asymmetry effects).

#### Multiple regression and correlational analyses

Step-wise multiple regression analyses were carried out. Dependent variables were only those EEG parameters that manifested significant sleep-related changes in a direction that was hypothesized to predispose an insight processing strategy. Accordingly, the normalized rate of change (in per cent) of occipital beta power, occipital alpha-2 power, right frontal beta power, and right frontal alpha-1 power were used as dependent variables in separate analyses. The independent variables were the rate of change after sleep (in percentage) of each of SP measures for each of the eight ROIs in each time window.

Sleep-related changes of EEG power were correlated by using Pearson correlation coefficient. The rates of EEG power changes (alpha-1, alpha-2, beta) at occipital electrodes were correlated with percentages of different sleep stages (S1, S2, S3, S4, REM sleep, and waking).

## Results

### Sleep Analysis

As reported for the slightly larger sample in ref. [16] (cf. Methods), sleep recordings confirmed the differential distribution of SWS vs. REM sleep. Subjects in the Early-NG had substantially more SWS than those in the Late-NG (26.6% vs. 9.6%, F(1/47) = 24.8, p<0.001), and subjects in the Late-NG, conversely, had substantially more REM sleep than those in the Early-NG (21.3% vs. 5.5%, F(1/47) = 80.8, p<0.001). The two groups did not differ in the proportions of other sleep stages (F(1/47)<1.9, p>0.2).

### Behavioural Results

Within the sample used, eight subjects from the Early-NG (32%) and 5 subjects from the Late-NG (21.7%) gained insight after sleep (χ^2^(1, n = 48) = 0.6, p = 0.4). Reaction times (RTs) were subjected to a Processing Strategy (Solvers vs. Non-solvers) x Sleep Group (Early-NG vs. Late-NG) x Session (pre-sleep vs. post-sleep) x Response Number (R1 to R7) ANOVA. RTs did not differ between the two sleep groups (all F(1/44)<1.5, p>0.15 for the main and interaction effects) nor between solvers and non-solvers (F(1/44)<0.4, p>0.7). RT significantly decreased after sleep (Session, F(1/44) = 9.8, p<0.005; mean RT before sleep = 1085 vs. mean RT after sleep = 1030 ms). This decrease was expressed for all response numbers (p<0.001 for each simple effect) with exception of R5 (F(1/44) = 0.4, p = 0.5). Being an immediate repetition of R4, R5 was the fastest response throughout.

Coefficients of RT variance (CV) were subjected to the same analysis. No significant effects of Session were found. Yet, only the solvers manifested a significant increase in CV after sleep, independently of whether they belonged to the Early-NG or Late-NG (Session x Processing Strategy, F(1/44) = 9.4, p = 0.005).

### Beta and Alpha EEG Power

#### Occipito-parietal spectral EEG

**Figure 3:**
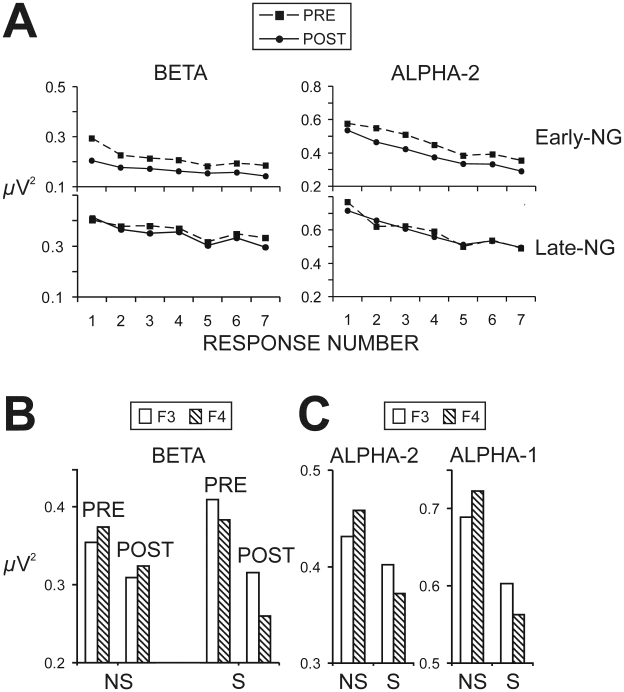
Spectral EEG measures. Power values are log_10_-transformed. (A) Power measures for beta and alpha-2 frequency bands at Oz and for consecutive response numbers (from R1 to R7) in the pre-sleep (PRE) and post-sleep (POST) sessions. Early-NG, early-night group; Late-NG, late-night group. (B) Frontal measures (F3, F4) of the spectral beta power for the non-solvers (NS) and solvers (S). PRE, pre-sleep session, POST, post-sleep session. (C) Frontal measures (F3, F4) of the spectral alpha (alpha-2 and alpha-1) power for the non-solvers (NS) and solvers (S) in the two sleep sessions pooled together.

Figure 3A shows that the occipital beta power decreased only after early-night sleep (F(1/23) = 16.5, p<0.0001), but did not change after late-night sleep (F(1/21) = 0.89, p = 0.3; Sleep Group x Session, F(1/44) = 4.5, p<0.05). Processing strategy had no effects. Beta power decreased with advancing string processing (Response Number, F(6/264) = 61.4, p<0.0001), yet independently of sleep group (Response Number x Sleep Group, F(6/264) = 0.4, p = 0.8) and condition (Response Number x Session, F(6/264) = 1.54, p = 0.2).

As illustrated in Fig. 3A, alpha-2 displayed the same effects as beta. It changed differentially after early- and late-night sleep (Sleep Group x Session, F(1/44) = 4.4, p<0.05), decreasing after early-night sleep (Session, F(1/23) = 4.89, p = 0.03), in contrast to late-night sleep (Session, F(1/21) = 0.053, p = 0.8). No effects of processing strategy were detected. As also found for beta, alpha-2 power decreased from R1 to R7 (Response Number, F(6/264) = 48.3, p<0.0001) in each group (Response Number x Sleep Group, F(6/264) = 1.3, p = 0.4) and sleep condition (Response Number x Session, F(6/264) = 0.34, p = 0.8).

Alpha-1 power increased after sleep (Session, F(1/44) = 4.58, p = 0.04) independently of sleep groups and processing strategy, and manifested the same decrease from R1 to R7 both before and after sleep (Response Number, F(6/264) = 13.07, p<0.001).

#### Asymmetric effects on spectral EEG at frontal recordings

Beta EEG power decreased after sleep in the two sleep groups (Session, F(1/44) = 22.5, p<0.001; Sleep Group x Session, (F(1/44) = 1.12, p = 0.3). As demonstrated in Fig. 3B, sleep-related beta decrease, however, was different for solvers and non-solvers (Processing Strategy x Session x Laterality, F(1/44) = 5.5, p = 0.02): beta decrease was significantly greater at the right (Processing Strategy x Session (F(1/44) = 5.5, p = 0.02) than at the left frontal site (Processing Strategy x Session (F(1/44) = 1.5, p = 0.2) in solvers relative to non-solvers. Mainly due to this differential reduction, there was a clear left > right asymmetry in solvers contrasting with the left<right tendency in non-solvers (Processing Strategy x Laterality (F(1/44) = 6.8, p = 0.01; Processing Strategy x Laterality after sleep (F(1/44) = 11.4, p = 0.002; Processing Strategy x Laterality before sleep (F(1/44) = 3.1, p = 0.08). The stronger post-sleep decrease of beta power on the right side in solvers was more expressed after early- than after late-night sleep (Sleep Group x Processing Strategy x Session x Laterality F(1/44) = 6.1, p = 0.017).

Figure 3A shows that alpha-2 EEG power decreased significantly after sleep (Session, F(1/44) = 17.3, p<0.0001; Session x Laterality, F(1/44) = 1.2, p = 0.3) but only in the Early-NG (Session x Sleep Group (F(1/44) = 7.25, p = 0.01; Session in the Early-NG (1/23) = 36.5, p<0.0001; Session in the Late-NG F(1/21) = 0.7, p = 0.4). As demonstrated in Fig. 3C, both before and after sleep, alpha-2 power was significantly smaller at the right frontal location in solvers than non-solvers from each group (Processing Strategy x Laterality (1/44) = 9.4, p = 0.004; Processing Strategy x Sleep Group x Laterality, F(1/44) = 2.2, p = 0.15; Processing Strategy x Session x Laterality F(1/44) = (1/44) = 0.2, p = 0.6) thus providing a marker for the subsequent insight in solvers.

As found for alpha-2, only the early-night sleep had an effect on alpha-1 power (Sleep Group x Session, F(1/44) = 4.9, p = 0.03; Early-NG, F(1/23) = 6.5, p = 0.017; Late-NG, F(1/21) = 0.3, p = 0.5). Figure 3C further shows that similar to alpha-2, alpha-1 power significantly differentiated solvers from non-solvers at the right frontal location both before and after sleep, independently of the sleep group and of the session (Processing Strategy x Laterality (1/44) = 4.8, p = 0.03; Processing Strategy x Sleep Group x Laterality, F(1/44) = 0.5, p = 0.45; Processing Strategy x Session x Laterality F(1/44) = 0.2, p = 0.65). As found for the occipital recordings, the three frontal spectral measures decreased from R1 to R7 both before and after sleep (p<0.001).

#### Asymmetric effects on spectral EEG at parietal recordings

For all spectral measures, power was significantly lower at P3 than P4 (Beta: F(1/44) = 4.1, p = 0.05; Alpha-2: F(1/44) = 10.9, p = 0.002; Alpha-1: F(1/44) = 3.26, p = 0.07). As found for occipital recordings, beta and alpha-2 decreased only after early sleep (Sleep Group x Session, (F(1/44) = 4.5, p = 0.04). Importantly, no main or interactive effects of Processing Strategy were detected for any of these spectral measures at parietal locations.

### Spatial Synchronization

**Figure 4:**
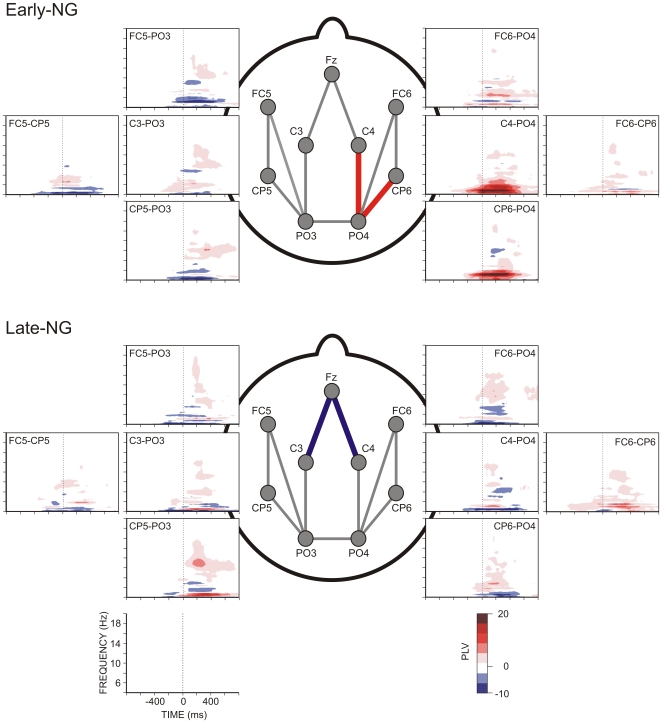
Spatial synchronization. Time-frequency plots of difference (post-sleep minus pre-sleep) phase-locking values (PLV) of selected pairs of electrodes from the left and right hemisphere for the two sleep groups, Early-NG and Late-NG. PLV increase after sleep is presented in red, whereas PLV decrease after sleep is presented in blue.

Figure 4 demonstrates differences between post- and pre-sleep spatial synchronization of EEG theta activity. To detail, the synchronization between left and right occipito-parietal regions (PO3-PO4) did not change after either early or late night sleep, nor was it affected by the processing strategy. However, for the phase-synchronization between parieto-occipital and centro-parietal regions, sleep induced a significant increase in the right-hemisphere for the Early-NG (PO4-C4, PO4-CP6: Z = −2.2, p = 0.03) whereas no significant effects were found for the Late-NG (p>0.5). In contrast, early-night sleep had no effect on phase-synchronization between frontal and motor (contra-lateral and ipsi-lateral) areas, whereas late-night sleep produced a significant decrease (Z = −2.7, p = 0.006; Z = −2, p = 0.05). Sleep-related changes in synchronization across sessions did not depend on Processing Strategy.

### Slow Potentials

#### Occipito-parietal slow potentials

**Figure 5:**
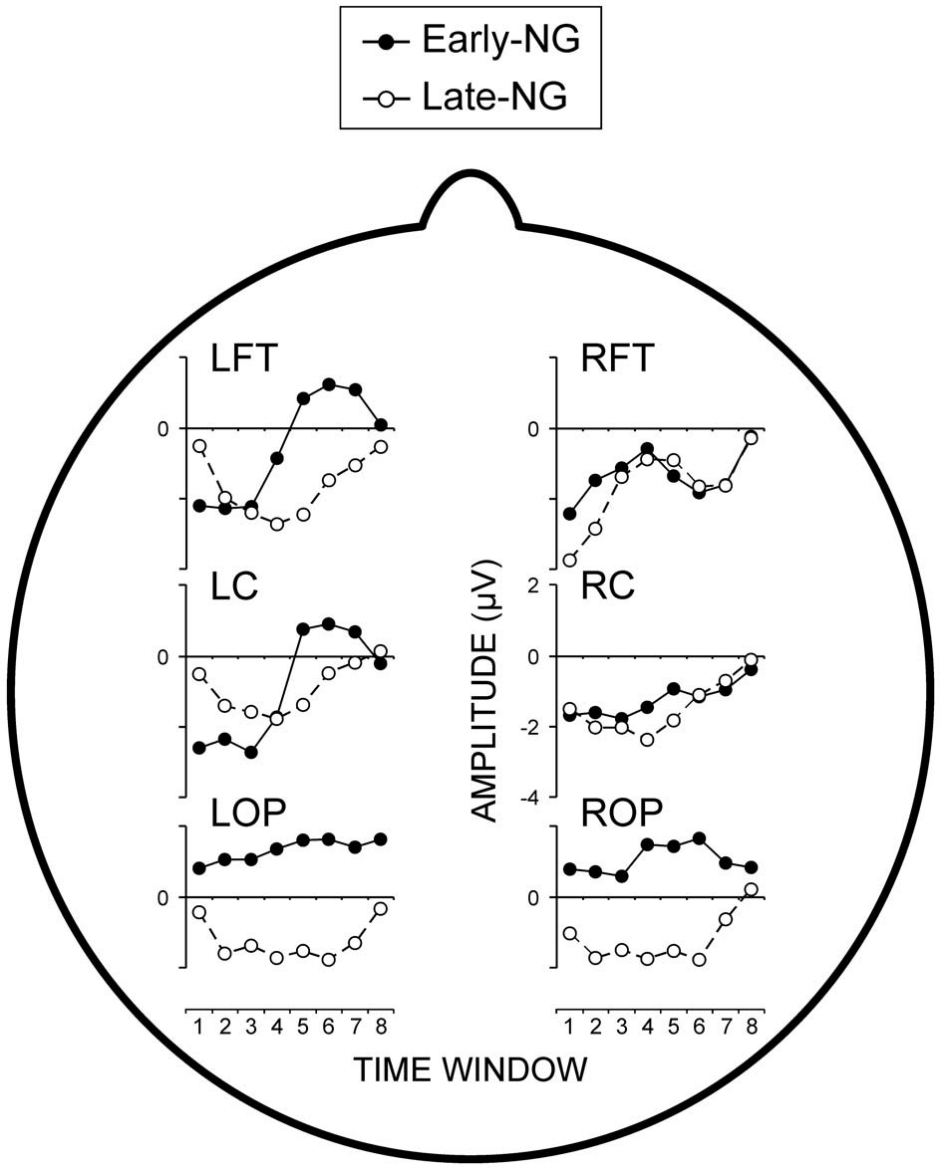
Slow potentials (SPs). Difference SPs obtained by subtracting pre-sleep from post-sleep SP measures in two sleep groups, Early-NG and Late-NG, for six regions of interest (LFT, left fronto-temporal, LC, left central, LOP, left occipito-parietal, RFT, right fronto-temporal, RC, right central, ROP, right occipito-parietal). Time windows 1 to 8 correspond to 1-s epochs of measurement, with the first one starting 1.5 s after string on-set.

Effects of sleep were similar for unpredictable and predictable responses and for LOP and ROP (Session x Predictability, F(1/44) = 0.2, p>0.6; Session x Predictability x ROI, (1/44) = 0.01, p>0.9). Yet, in the Early-NG, occipito-parietal SPs tended to decrease (become more positive) after sleep indicating a reduction in functional activation, whereas in the Late-NG, SPs tended to increase (become more negative) after sleep indicating a stronger functional activation within the visual system. This differential effect of early vs. late sleep was captured statistically by difference SP measures obtained by subtracting pre-sleep from post-sleep SP values (Sleep Group, F(1/44) = 6.4, p = 0.015, Fig. 5).

#### Asymmetric effects on slow potentials

Figure 5 further illustrates that after late sleep, the values of difference measures were negative indicating that late sleep led to an overall negativization of SPs at the two hemispheres. This negativization was also similar for the unpredictable and predictable responses (Laterality x Predictability, (F(1/21) = 1.29, p>0.3; Laterality x Predictability x ROI, F(2/44) = 1.42, p>0.25). In contrast, early-night sleep produced a negativization at the two hemispheres for unpredictable responses, whereas for predictable responses, SPs became more negative only over the right hemisphere (Laterality x Predictability, F(1/23) = 4.2, p<0.05). This difference is also verified by the significant Sleep Group x Laterality x Predictability interaction (F(1/44) = 4.61, p<0.05) and tended to disappear at parieto-occipital regions as reported above (Laterality x Predictability x ROI x Sleep Group, F(2/92) = 2.56, 0.1>p>0.05). Thus, hemispheric asymmetry did not change for either the unpredictable or predictable responses after late-night sleep, whereas the right hemisphere appeared more activated for predictable responses after early-night sleep (Fig. 5). No significant main or interactive effects of Processing Strategy were found.

### Correlations of Spectral EEG Power with Sleep Stages

Table 2 presents the results of correlation analyses testing the associations between sleep-related changes in spectral EEG, and the amount of sleep stages. There were significant negative correlations between the decrease of both alpha-2 and beta EEG power at occipital locations after early sleep and SWS, meaning that more SWS (S3, in particular) was associated with a greater occipital reduction of alpha-2 and beta. No significant correlations were found between other spectral measures and sleep stages.

**Table 2:** Results of correlational analyses. Significant correlations between amount of sleep stages and occipital spectral EEG in the early-night group (n = 24).

Variables	Pearson r	*P*
**beta:** electrode PO4 - S3 (%)	−0.42	0.03
**alpha-2:** electrode PO4 - S3 (%)	−0.39	0.05

beta, EEG beta power; alpha-2, EEG alpha-2 power; PO4, right parieto-occipital electrode; S3, sleep stage 3; *P*, *P*-value.

### Correlations of Spectral EEG Power with Slow Potentials

Sleep-related changes in occipital spectral measures and in SPs were found only after early-night sleep. This raises the question if these alterations were interrelated. To explore this question, a multiple regression analysis was performed, in which the dependent variables were the occipital spectral measures reflecting the change of the parameter from before to after sleep. The independent variables were the rates of change in SPs from all time windows and all regions of interest. The major assumption was that in case of interdependence, a solution of a model would be found that would select time- and region-specific SP predictors of sleep-related changes of occipital EEG. Table 3 shows that for both the rate of change of occipito-parietal beta and alpha-2 after early-night sleep, the rate of change of the left temporo-frontal SPs from the second half of string processing was modeled as a significant predictor.

**Table 3:** Summary of multiple regression analysis (MRA).

No.	Dependent variable	R	R^2^	R^2^adj	F(1/22)	*P*	Prediction	B	β	t	*P*
1.	Rate of occipital EEG beta power change	0.496	0.246	0.212	7.18	0.014	Constant LFT (TW-5)	−14.800 0.066	−0.50	−2.85 2.68	0.009 0.014
2.	Rate of occipital EEG alpha-2 power change	0.512	0.263	0.229	7.80	0.010	LFT (TW-5)	0.088	0.52	2.80	0.010

Significant SP predictors of occipital EEG spectral measures are presented only. MRA parameters: R, multiple correlation coefficient (between observed and model-predicted values); R^2^, coefficient of determination; R^2^adj, adjusted coefficient of determination (compensates for model complexity); B, regression coefficient; b, standardized coefficient for the predictor.

F, t, *P*, ANOVA parameters; LFT, left fronto-temporal region; TW-5, time window 6.5–7.5 s during predictable response processing.

### Subjective Ratings

Ratings of subjective feelings of sleepiness, activation, tension, boredom, motivation, and concentration were obtained before and after each session of initial practice and retest. In line with the results reported in Yordanova et al. (2008) [16], the present subsamples did not differ on the whole in these variables, as indicated by non-significant main effects of early vs. late night (all p>0.2). In the two sleep groups, subjects felt more sleepy and less activated, motivated and concentrated in task sessions performed in the middle of the night (i.e., initial practice for Late-NG, retest for Early-NG) than in sessions performed in the evening (initial practice for Early-NG) or in the morning (retest for Late-NG) (p<0.05, for Sleep Group x Session interactions). This effect was, however, much weaker than an activating effect of task performance itself independent of the time of the session, i.e., subjects felt less sleepy and more activated at the end as compared to the beginning of a task session (p<0.001).

## Discussion

Specific functional activations of the brain can increase the probability of solving problems with insight in tasks with verbal anagrams. The mediating mechanisms have been associated with less selective attention in the occipital visual cortex and an asymmetric right-hemisphere activation [7], [9]–[10], [27]–[28], [49]. The present study explored (1) if such activations may be induced by sleep, (2) if their appearance may be affected differentially by early-night sleep, rich in SWS, and late-nigh sleep, rich in REM sleep, (3) if they may promote the gain of insight after sleep in the number reduction task [14], [16], and (4) if they depend on the processing of task-specific information in the NRT, or may emerge as non-specific post-sleep functional activations.

One major result was that functional activation patterns linked with the insight mode of processing [27]–[28] were induced by early-night sleep but not by late-night sleep. Changes included a reduction of occipital beta and alpha-2 spectral EEG power accompanied by a widespread decrease of alpha activity, and an increase in the spatial synchronization of the right parieto-occipital association cortex indicating increased functional connectivity only in the early-night group [52]–[53], [73]. Yet, these changes were not associated with a higher rate of insight to NRT regularity after early sleep. Instead, as the second major result of the present study shows, a reduction of spectral beta and alpha activity at the right frontal region appeared as a specific functional marker of insight in the NRT, since this effect was detected only in those subjects who would subsequently gain insight to the hidden regularity of the task. Notably, both the SWS and REM sleep produced an increased right frontal asymmetry in subsequent solvers. Thus, only the early-night sleep modified functional activation patterns of occipito-parietal regions, but the critical determinant of post-sleep insight to NRT regularity was the right frontal activation, which was enhanced by sleep only in subsequent solvers independently of the predominant sleep stage.

The gradual reduction of beta and alpha power at occipital locations across single string processing indicates a dependence on response sequence. This reduction appears as a specific feature of NRT processing by being present both before and after sleep, early and late. Previously, a BOLD signal at the superior parietal cortex has been found to increase toward the end of string processing in the NRT irrespective of whether a hidden mirror rule was incorporated in the string or not, reflecting a fast short-term proceduralization of stimulus- response associations [74]–[75]. Likewise, a progressive decline of alpha power maximally expressed at the central cortex contra-lateral to movement has been correlated with implicit learning of structured motor sequences [72], [76]. With regard to these observations, currently observed effects of response sequence imply that in the beginning of each string processing, visual attention is more strongly focused to support computational demands [77]–[78]. With advancing sequence, a short-term proceduralization occurs and modifies attention from focused to automatic [74]–[75], which facilitates (reduces) the maintenance of active processing of task-specific elements [41] and enhances non-specific diffuse activations. These observations from the NRT substantiate previously found associations between occipital beta and alpha activity and attentive visual processing [27]–[28].

With this account, spectral EEG changes across sleep point to a reduction of selective visual attention after early-night sleep. Although various neurohumoral, neurotransmitter and circadian variatons in the neural mechanisms known to accompany SWS dominance in the first half of the night and REM sleep dominance in the second half of the night may contribute to the observed effects, currently found associations with the amount of SWS emphasize the specific role of SWS for changes in spectral EEG parameters at occipito-parietal regions. Of note, the reduction of selective visual attention after early-night sleep reflected by spectral EEG was similar for all response types indicating a state-dependent rather than a task-specific effect. This effect cannot be associated with the activation of explicit processing systems since none of the subjects had discovered the abstract mirror principle during the first block after sleep analyzed here. Nor can these changes reflect a mere behavioural speeding because RT decrease after sleep was similar for the early- and late-night groups. Ratings of subjective feelings also did not differ between the groups, and overall performance variance did not change after sleep nor did it either differ between the early- and late-night groups. Thus, gross arousal/attention variations may not account for the spectral EEG changes after early sleep.

Instead, changes in occipital spectral measures after early sleep were predicted by the sleep-related changes in left temporal-frontal negative potentials associated with the implicit processing of predictable items (Table 3). Hence, the accumulation of implicit knowledge before sleep or the consolidation of implicit task representations during sleep [16], [18] appear to provide a critical prerequisite for the changes in occipital functional states after early sleep. Notably, item non-specific positivization of parieto-occipital slow potentials indicating reduced controlled visual processing [18], [57] was also found here only after early sleep. Altogether, these correlational results imply that SWS dominating in the first half of the night basically supports off-line learning within the visual system, which confirms previously reported specific associations of early- but not late-night sleep with improvement in visual discrimination skills [79]. Further, these results point to co-existent functional alterations of state-dependent and task-dependent activation patterns in the visual system as reflected by spectral EEG measures and slow cortical potentials, both of which are co-supported by SWS. Finally, the present results indicate that sleep-related alterations in functional visual states are induced by a reorganization of implicit task representations during SWS [18] and are therefore determined by information-specific mechanisms.

Importantly, insight to the hidden regularity of the NRT after sleep, early or late, was associated with activation of the right frontal region. Previously, visuomotor tasks have been found to differ in the reduction of alpha and beta activity at dorsolateral frontal areas depending on the required degree of cognitive control, which reflected a difference in the spread of task-relevant information across frontal areas [80]. According to the present results, sleep induced a decrease of spectral beta and alpha-2 activity at right frontal areas but only in those subjects who subsequently gained insight into the NRT. Of note, solvers manifested signs of greater right frontal involvement already before sleep. This confirms the role of right-hemisphere activation for promoting insight also in the NRT [27]–[28]. Further, evidence is provided that sleep enhances the insight-related activation of the right frontal areas. Yet, a specific contribution of different sleep stages (SWS and REM sleep) in these mechanisms cannot be delineated with the measures used here. Nor can it be excluded that individual traits or specific processing modes at pre-sleep learning could have promoted subsequent insight, irrespective of whether sleep occurred or not in the retention interval [17]. It is, however, notable that increased post-sleep synchronization indicating a stronger connectivity of the parieto-occipital regions of the right hemisphere was found here only after early-night sleep. Given the role of right parieto-occipital regions for visual awareness [81]–[84], and the role of right frontal activation for bringing hidden task regularity to awareness as described above, an increased signaling within the right hemisphere may be involved in a mechanism that activates explicit processing systems or promotes the transformation of implicit knowledge into explicit [16]. According to the results, SWS may contribute to this mechanism by facilitating the connectivity and information transfer within the right hemisphere.

### Conclusions

It is concluded that changes in functional activation patterns during NRT performance occur only after early- but not after late-night sleep. These changes are associated with a decrease in controlled processing within the visual system and with an increase in the functional connectivity of the right hemisphere, and are supported by SWS in the first half of the night. Insight to the hidden NRT regularity is coupled with right frontal activation, thus being specifically prone to potentiation by SWS.
